# Characterization of Kenyan Honeys Based on Their Physicochemical Properties, Botanical and Geographical Origin

**DOI:** 10.1155/2019/2932509

**Published:** 2019-01-10

**Authors:** Mary Wanjiru Warui, Lise Hansted, Mary Gikungu, John Mburu, Geoffrey Kironchi, Aske Skovmand Bosselmann

**Affiliations:** ^1^Department of Land Resource Management and Agricultural Technology, University of Nairobi, P.O. Box 29053-00625, Nairobi, Kenya; ^2^Danish Beekeepers Association, Fulbyvej 15, Sorø, 4180, Denmark; ^3^National Museums of Kenya, P.O. Box 40658- 00100, Nairobi, Kenya; ^4^Department of Agricultural Economics, University of Nairobi, P.O. Box 29053-00625, Nairobi, Kenya; ^5^Department of Food and Resource Economics, University of Copenhagen, Rolighedsvej 25, 1958 Frederiksberg. C, Denmark

## Abstract

Properties and composition of honey are essential in providing information regarding their quality as well as in their differentiation based on production region characteristics, e.g., floral sources. This paper presents physicochemical properties and floral sources (botanical origin) of 21 honey samples obtained from arid and semiarid areas of Kenya, specifically, West Pokot, Baringo, and Kitui Counties. Physicochemical parameters which were analyzed to determine honey quality included moisture content, hydroxymethylfurfural (HMF), diastase activity, free acidity, and electrical conductivity. Values of these parameters were compared with those of the existing local, regional, and international standards for honey. Melissopalynological analysis (pollen analysis) was also carried out to provide information on botanical origin of the honeys. Results showed mean parameter values of moisture, 16.34%; HMF, 23.28 mg/kg; diastase activity, 10.67 Schade units; free acidity, 22.95 meq/kg; and electrical conductivity, 0.40 mS/cm. Free acidity and electrical conductivity values of honey samples obtained from West Pokot were significantly lower than the values of honeys from Baringo and Kitui. Eighteen (18) honey samples had all parameter values within the limits set in the East African, Codex Alimentarius, and the European Union directive standards for honey. Results also showed a total of 29 pollen types in the honey samples analyzed, and* Acacia spp*. was the predominant pollen type in 4 of the 21 honey samples. Findings of this study showed that Kenyan origin honeys can tap into the existing regional and international markets based on their quality which can be attributed to their botanical origin. Results of this study also suggested that honey producers have undertaken appropriate measures in honey harvesting, processing, handling, and storage. However, there is a need to build capacity of producers whose honey were of unacceptable quality. This would involve training on proper honey production, processing, and handling practices as well establishment of honey collection and processing centres at the local level in order to improve honey quality. This will enhance access to existing honey markets. Conservation of bee floral sources would also be needed to maintain honey quality.

## 1. Introduction

Honey quality and characteristics are greatly influenced by natural factors (e.g., climatic conditions and floral sources which provide nectar and pollen) and human factors (e.g., traditions and know-how of producers in harvesting periods and methods, handling, processing, and storage techniques) [1–8]. Substances which determine honey quality include pollen, water content, sugar content, proteins, enzymes, phenolic compounds, flavonoids, vitamins, minerals, organics acids, solid particles, and free amino acids [9, 10]. Honey also has a specific flavour and aroma based on the floral source [11]. Composition and properties of honey are known to vary across different regions [12, 13].

The quality of honey can be determined through physicochemical analysis, melissopalynological analysis, and sensory analysis [14–16]. These analyses provide useful information which can be used to verify authenticity of honey as well as its botanical and geographical origin [16–18]. Among the quality parameters considered in honey trade are physicochemical parameters (moisture, Hydroxymethylfurfural (HMF), diastase activity, electrical conductivity, free acid, sugars, and water insoluble contents). Levels or values of these parameters in a honey indicate its quality. In European countries, botanical origin of honey influences its prices in the market and demand by consumers.

In Kenya, honey production is concentrated in arid and semiarid lands (ASALS) [19]. Honeys from ASALS were identified as having potential for protection with Geographical Indication (GI) (an indication which links honey quality/characteristics to its geographical origin) [20]. This demonstrates the capacity of Kenya's honey subsector to tap into world GI markets which can create additional monetary values from higher value added honeys based on geographical origin attributes, which can be linked to human factors as well as the natural environment. Among the identified honeys were Kitui, West Pokot, and Baringo honeys. The Kenya Honey Council (KHC), a honey producer association in Kenya and other actors in the honey subsector, envisions to expand markets and value for these honeys through GI protection [21] so as it can enhance rural livelihoods, economic development, and thus sustainable development. However, information on the quality traits and botanical origin of these honeys is scarce. Such information is important in enhancing consumer confidence on quality of a product and its traceability, thereby enhancing honey trade at the local level and beyond including the GI markets. Therefore, the aim of this study was to (i) assess the physicochemical properties of West Pokot, Baringo, and Kitui honeys in order determine if they are of acceptable quality according to national, regional, and international market; (ii) assess differences between honeys from the three study areas based on physicochemical properties; and (iii) determine the botanical origin (floral sources) of the honeys from the three regions. Results of this study will demonstrate whether the Kenyan origin honeys can tap into the existing markets and niche markets based on their quality which can be differentiated from that of other honeys produced in other regions, thus developing GI honey.

## 2. Materials and Methods

### 2.1. Honey Sample Collection

**Figure 1:**
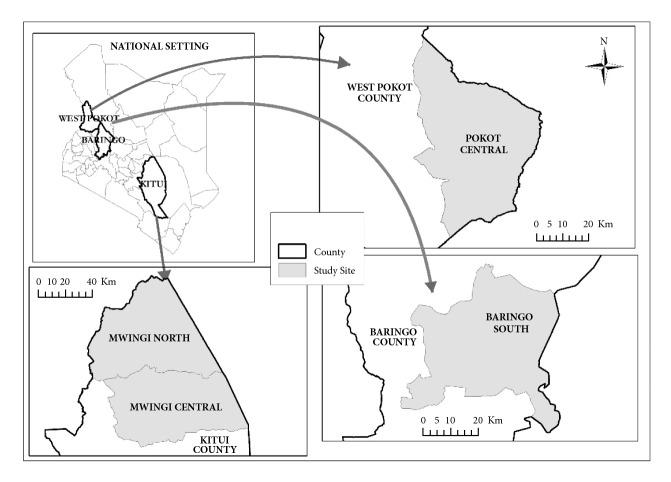
A map showing areas where honey samples were obtained.

Honey samples harvested in the first season of the year, i.e., between February and May 2015, were obtained from beekeepers in lowlands of West Pokot, Baringo, and Kitui Counties, Kenya (Figure 1). These areas are categorized as arid and semiarid areas, and rain-fed agriculture in these areas is unreliable; thus, beekeeping is largely practised and it is a viable venture. Honey production in the three study areas is an important source of livelihood for the community [22–24]. Vegetation in the three areas where the honey samples were collected is mainly of natural forests comprising shrubs and woody or thorny trees, with* Acacia spp* dominating the regions [25, 26].

Seven honey samples were collected in each study area, totalling 21 samples. Each sample was placed in a fresh, sterile, closely tightened food grade container. Containers with the honey samples were labelled with a number, place, and date of collection for easy identification. The samples were stored at room temperature (25°C) and kept away from direct sunlight and moisture in order to ensure that their quality as per the time of collection was maintained. Analysis of the honey samples was conducted at Quality Services International (QSI) GmbH in Bremen, Germany.

### 2.2. Physicochemical Analysis

Physicochemical analysis for measurement of moisture content, HMF, diastase activity, free acidity, and electrical conductivity in each honey sample was done based on methods described by the Association of the Official Analytical Chemists [27] and harmonized methods of the International Honey Commission [1, 28].

Moisture is an important parameter in determining honey quality. Too high moisture content often causes fermentation of honey leading to low shelf life and unpleasant flavour [29]. Climatic conditions, degree of honey maturity reached in the hive and handling during harvesting, processing, and storage all determine the level of water content in honey [30, 31].

The level of HMF indicates freshness of honey and it is affected by the storage conditions/periods and the extent to which it is heated (Küçük, 2007) [32, 33]. HMF level in honey is also influenced by moisture content and presence of organic acids and sugars. High HMF levels in a honey sample suggest a possibility of adulteration, overheating, or storage for a long period of time [34, 35].

Diastase is a natural enzyme which occurs in honey and is sensitive to heat. Low levels of the enzyme in the honey indicate excessive heating during processing and/or storage for too long or under high temperatures [29, 36, 37]. Diastase content in the honey also depends on floral sources, nectar collection period by a bee colony, and its flow.

Acidity in the honey is influenced by organic acids, particularly gluconic acid which emerges from bacterial action and glucose activity during honey ripening [38, 39]. Free acidity influences honey flavour, texture, shelf life, and stability [40].

The electrical conductivity in honey is linked to concentration of organic acids, mineral salts, ash, complex sugars, and proteins [41]. This honey parameter varies depending on floral origin, and it is important in classifying honey as from either nectar or honeydew [42].

To ascertain if the physicochemical parameter values obtained from the analyzed honey samples were of acceptable quality as required in national, regional, and international markets, the physicochemical parameter values were compared with the recommended levels as specified in the East African Standards (EAS) for honey (EAS 36:2000), Codex Alimentarius honey standards (CODEX STAN 12-1981), and the European Union (EU) directive for honey (2001/110/EC).

### 2.3. Melissopalynological (Pollen) Analysis

Melissopalynological analysis was undertaken to determine the botanical origin (floral sources) of the honeys based on pollen types in the honeys. Identification of the pollen types in honey samples and determination of their relative frequencies in each sample were done using the methods described by Louveaux et al. [43], Louveaux et al. [44], Feás et al. [45], and Yang et al. [46]. Relative frequencies of identified pollen types were determined through grouping and counting pollen grains in the prepared sediment of each honey sample [18] using a microscope. Pollen types in the honeys were useful in determining the botanical and geographical origin of the honeys. Confirmation of geographical origin of honey was based on the identified pollen spectrum being consistent with the flora of the particular region from where honey samples were obtained [15, 29, 44].

### 2.4. Statistical Analysis

Means and standard deviation of the physicochemical property values for the honey samples collected in each study area as well as overall mean for honeys samples from all the study areas were calculated. Analysis of Variance (ANOVA) was used to determine the differences in physicochemical properties parameters values of honeys obtained from the three study areas. Relative frequencies of each pollen type in each honey sample were calculated and expressed as percentages based on total number of pollen grains counted. Pollen identified in the honey samples were categorized as predominant pollen types (>45%); secondary pollen types (16-45%); important minor pollen types; (3-15%) and minor pollen (<3%) [15, 43, 44].

## 3. Results and Discussion

### 3.1. Physicochemical Properties of Sampled Honeys

The physiochemical parameter values of the honey samples from the three study areas, and their mean and standard error are summarized in Table 1. Mean values of physicochemical parameters are compared across the three study areas.

The moisture content of honey samples from the three study areas ranged from 14.20 to 17.40%, with an overall mean of 16.34±0.88%. These values were below the maximum limit of 22% as recommended by EAS and 20% as recommended by Codex and EU directive for honey. The results indicated that the honey producers in the study areas harvested ripe and capped honey and that they stored honeys under suitable condition where moisture could not be absorbed. There was no significant difference in moisture content percentage between honey samples obtained from the three areas. However, moisture content of honey samples from Kitui was higher than that of honeys from West Pokot and Baringo. Variation in moisture content can be attributed to botanical origin of the honeys [31] or the weather conditions during production of the honeys.

Hydroxymethylfurfural (HMF) of the honey samples analyzed ranged between 11.00 and 120.00 mg/kg. The HMF value of one honey sample which had 120 mg/kg was marked as an outlier and, thus, was not used in calculation of mean and statistical differences for the honey parameter values. The high HMF value of this honey sample exceeded the maximum limit value specified in EAS, Codex standard, and EU directive for honey. This result suggests faulty processing and storage conditions. The overall HMF mean ± SE for the honey samples was 23.28±9.10 mg/kg and there was no significant difference in HMF content between the three study areas.

Diastase activity values of the honeys analyzed ranged between 2.70 and 17.50 Schade units (10.67±3.34 Schade units). Although there was no significant difference in diastase activity value between honeys from the three areas, honeys obtained from Kitui had a lower mean diastase activity as compared to the other two areas. Diastase activity values of three honey samples from Kitui (samples 17, 20, and 21) were below the minimum limit of 8 Schade units as specified by Codex and EU directive for honey, while only one honey sample (21) was below the minimum limit of 3 Schade units as specified by EAS. This explains the lower diastase activity mean for honey samples from Kitui. These results indicate that the three honey samples had low enzyme content resulting from overheating of the honeys or storing them under high temperatures.

Free acidity of the honeys ranged between 17.00 and 29.00 meq/kg (22.95±4.33 meq/kg) and these values were below the maximum limit of 40 meq/kg as stipulated by EAS and 50 meq/kg as specified by Codex and EU directive for honey. These results indicated that the honeys were ripe during harvesting and they had low water content and thus absence of fermentation [47]. Free acidity values of honey samples obtained from West Pokot were significantly lower than the values of honeys from Baringo and Kitui and this can be attributed to differences in sugar concentration of the nectar [38]. This is associated with variation of botanical (floral) sources between the three areas as evidenced in other studies by Babarinde et al. [48], Sahinler & Gul [49], and Williams et al. [50].

Electrical conductivity of the honey samples ranged between 0.26 and 0.59 mS/cm (0.40±0.10 mS/cm). These values were below the maximum value of 0.8 mS/cm as specified in Codex and EC directive. These results indicated that the honeys were either blossom honeys or blends of blossom and honey dew honeys [1, 41, 42]. EAS does not specify the required level of electrical conductivity in honeys. Electrical conductivity values of honey samples derived from Baringo and Kitui were significantly higher as compared to electrical conductivity values of those obtained from West Pokot. These results can be attributed to high acidity values of Baringo and Kitui honeys [51, 52] or the variation in geographical and botanical origin of the honeys.

In summary, 18 honey samples (all samples from West Pokot and Baringo and 4 from Kitui) had all physicochemical parameter values within the acceptable limits as stipulated in the EAS, Codex, and the EU directive for honey. The 18 honeys were classified as table honeys (i.e., honeys whose quality is fit for direct human consumption). Results indicated that producers and processors of these honeys take appropriate measures to safeguard its quality. These measures include harvesting mature/ripe honey, use of appropriate temperatures while processing honeys, and storing honeys under favourable conditions (i.e., areas free from moisture and high temperatures). Results of the 18 honey samples indicate that the honeys are acceptable and can gain reputation in local, regional, and international markets, including the EU where honey demand is highest.

Results of the other 3 honey samples from Kitui, with unacceptable HMF and diastase activity levels, were classified as industrial honeys (i.e., honey of lower quality, thus used only in industries). This was an indication that some producers/processors did not take deliberate measures when processing/handling/storing honey. The low quality of the honeys can negatively influence their authentication and reputation, thereby their demand and market scope. Production of low quality honeys in Kitui could be a result of limited capacity and lack of implementation of regulations governing honey production, processing, packaging, and storage. Also, findings from a study conducted at a honey processing and marketing centre in Kitui, i.e., Mwingi honey market place, where the three honey samples were collected revealed that honeys of uncertain quality were sourced from brokers and other individual producers outside the production region due to low production of honeys by the authorized producers within the study region [53]. Since results of this study showed that some of the honey samples from Kitui were of good quality, it could be likely that the problem in the low quality honeys is as a result of poor processing, handling, or storage by producers and brokers where the honeys were sourced by the processing centre.

### 3.2. Botanical and Geographical Origin of Sampled Honeys

Results of melissopalynological analysis are presented in Table 2. A total of 29 pollen types were identified from the 21 honey samples analyzed. Of these pollen types, 11 were identified as of family level, 17 to genus level, and only one to species level. Overall, 21 pollen types were recorded in honey samples from West Pokot and Kitui and 20 pollen types in samples of Baringo honeys.

The results of this study showed that naturally occurring plants, e.g.,* Acacia spp. *and* Brachystegia spp.*, as well as introduced/cultivated plants, e.g.,* Trifolium spp., Bidens spp*.*, Eucalyptus spp., Prosopis spp.*, Euphorbiaceae, Combretaceae, Brassicaceae,* Zea mays, *and* Coffea spp., *are floral sources for bees. This observation agrees with findings of other plant-pollinator interaction studies where observations of bees foraging on multiple plant species from both natural and agricultural ecosystems were made [54, 55] and different pollen spectrum was observed in honeys [56, 57].

Four honey samples (1-4) from West Pokot having* Acacia spp*. as a predominant pollen type could be classified as unifloral honeys. Similar findings have earlier been found by Gloor [23]. The predominance of Acacia pollen type in the four honeys is likely attributed to honey bees preference for* Acacia spp.* and the floral rewards by the preferred plant [54] as well as its massive flowering in West Pokot during the study season.* Acacia spp*. has numerous flowers per inflorescence [58], which provide great floral reward (pollen/nectar) to its visitors. The results showed that other 17 honey samples had a combination of secondary pollen, important minor pollen, or minor pollen and therefore could be classified as multifloral honeys. This result could be attributed to honeybee preference for specific flora based on its availability in a specific region, as well as limited/less frequency of honey bee visits to flowering plant species in a particular region [56].

Some pollen types were recorded in all or most honey samples obtained from West Pokot, Baringo, and Kitui despite the differences in geographical location of the three areas. This is an indication of similarities of vegetation types in the study areas based on agroecological zonation. The common pollen types included* Acacia spp., *Euphorbiaceae, Combretaceae,* Brachystegia spp., Bidens spp., *Capparaceae, Poaceae (grasses),* Caesalpinia spp., *Sapindaceae,* Leucaena spp*., and* Triumfetta spp. *Four of these pollen types* (Acacia spp., *Euphorbiaceae, Combretaceae, and* Brachystegia spp.) *were recorded in all honey samples in varying quantities. Some pollen types were observed in honey samples obtained from specific areas and they included* Prosopis spp*. in Baringo,* Eucalyptus spp.* in West Pokot,* Olea spp.*, Apiaceae,* Taraxacum spp., Zea mays*, and* Trifolium *sp. in Kitui. Representation of specific and common pollen types in the honey samples analyzed is attributed to distribution and diversity of plants in particular area depending on the ecological/ecoclimatic zone of an area [25, 26].

Presence of pollen types of cultivated plants, i.e.,* Coffea spp. *and* Zea mays*, in the honey samples implied that agroecosystems provide bee floral sources. Plants from such ecosystems can be useful in supporting bees survival and production of honey especially when the natural flowering plants are not blooming. Similar observations were noted in studies conducted by Gikungu [54] and Karanja et al. [59]. More so, some cultivated crops which are specific in particular regions have been noted to yield unique honeys in EU whose attributes have facilitated its protection with GI (EU DOOR Database). Therefore, management of agroecosystem in the study areas can support survival of bees and yield honey with characteristics which can enhance their GI potential and sustainable production.

The results further showed pollen types of vegetation present in the three study areas, including those dominant in arid and semiarid areas [23, 60–62]. These results confirm the geographical origin of the honeys, differences in honey pollen composition across regions, and the natural link to honey characteristics; thus this can form a basis for protection of the three honeys with geographical indications, which identifies a product originating from a territory or a region where a given quality and other characteristics of the product are exclusively or essentially attributable to its geographical origin.

## 4. Conclusion

Results of this study showed that honeys from West Pokot, Baringo, and Kitui are of good quality and they generally meet the standards for local, regional, and international market, particularly EU. Melissopalynological analysis showed differences of the pollen spectrum between honeys from the different study areas based on geographical zones. Pollen types represented in the honeys were typical to vegetation of the study areas. Compliance of the honeys to the existing honey standards as well as the natural link to honey quality can form a basis for selling the product in niche market and in its protection with GI. To improve the low quality honeys and maintain the ones with good quality as well as enhance their link to natural and human factors, there is a need for development of the honey value chain. Activities/initiatives which can facilitate this include training of honey producers and processors on best production practices and conservation of bee flora, quality assurance of honeys at the local level, and establishment of local infrastructure (e.g., honey processing and marketing centres and road). To improve the quality of Kitui honey, development of a monitoring plan would be necessary. This tool would enhance honey traceability, control of the honey value chain. Also, capacity building of producers and processors on appropriate production, handling, processing, and storage techniques as well as establishment of collection centres and other infrastructure, e.g., roads, would be needed. Development and implementation of policies and laws to support honey production and marketing are also needed.

## Figures and Tables

**Table 1:** Physicochemical results of the honey samples obtained from the three study areas.

**Study Area **	**Honey** **Sample**	**Moisture content (**%**)**	**HMF (mg/kg)**	**Diastase activity** **(Schade units)**	**Free acidity** **(meq/kg)**	**Electrical conductivity (mS/cm)**
**West Pokot**	1	15.70	19.00	8.90	18.50	0.30

	2	15.80	19.30	9.60	18.50	0.31

	3	15.80	27.30	8.80	17.00	0.26

	4	15.80	26.80	8.60	17.00	0.26

	5	15.80	23.30	12.50	18.50	0.28

	6	15.80	23.10	9.90	18.50	0.28

	7	16.90	44.00	17.50	20.00	0.44

	**Mean±SE**	**15.94±0.42 a**	**26.11±8.52 a**	**10.83±3.23 a**	**18.29±1.04 a**	**0.30±0.06 a**

**Baringo **	8	15.00	11.00	12.60	25.50	0.56

	9	14.20	23.90	9.50	25.50	0.54

	10	17.40	28.90	9.30	28.50	0.44

	11	17.40	28.90	11.70	29.00	0.44

	12	17.40	28.40	10.60	29.00	0.44

	13	15.70	22.60	13.60	29.00	0.59

	14	16.00	15.00	12.40	22.00	0.42

	**Mean±SE**	**16.16±1.29 a**	**22.67±7.15 a**	**11.39±1.63 a**	**26.93±2.70 b**	**0.49±0.07 b**

**Kitui**	15	17.10	17.10	8.60	24.00	0.38

	16	16.80	11.30	14.50	25.00	0.44

	17	16.60	35.50	7.50	20.50	0.39

	18	17.40	11.80	16.20	29.00	0.52

	19	16.70	11.80	12.20	25.00	0.45

	20	16.70	36.60	6.80	20.00	0.38

	21	17.20	120.00*∗*	2.70	22.00	0.35

	**Mean±SE**	**16.93±0.30 a**	**20.68±12.10 a**	**9.79±4.74 a**	**23.64±3.12 b**	**0.42±0.06 b**

**P value**		*0.081*	*0.575*	*0.683*	*0.004*	*0.001*

Means with same letters within a column are not significantly different (P<0.05).

*∗*= The HMF value was not used in determining Mean and statistical differences for the honey sample parameter values since it is an outlier.

**Table 2:** Percentages of pollen types in West Pokot, Baringo and Kitui honey samples.

Pollen type	Pollen type percentage (%)																				
	**West Pokot** (n=7)							**Baringo** (n=7)							**Kitui** (n=7)						
*** Honey Sample***	*1*	*2*	*3*	*4*	*5*	*6*	*7*	*8*	*9*	*10*	*11*	*12*	*13*	*14*	*15*	*16*	*17*	*18*	*19*	*20*	*21*
*Acacia spp.*	50	54	58	48	37	42	9	37	25	8	11	13	10	20	15	4	17	11	17	15	4

Euphorbiaceae	7	10	17	12	12	14	2	m	m	m	m	m	m	m	18	18	m	16	19	m	m

Combretaceae	m	7	5	7	9	10	18	m	21	35	26	35	27	m	17	23	6	20	26	11	32

*Brachystegia spp.*	m	m	m	m	m	m	m	m	m	m	m	m	m	m	m	m	m	m	m	m	m

*Bidens spp.*	16	20	12	16	10	6	19	13	m	7	3	m	8	4		20	3	m	8	3	36

Capparaceae	3	m	m	m	m	m	m	m	12	20	30	20	11	2	11	10	m	m		m	m

Poaceae	m	m	m	m	m	m		m	m	m	m	m	m	m	m	17		m	m	m	m

*Caesalpinia spp.*	m	m	m	m	m	m	m	m	m	m	m	m	m		m	6	m	m	3	m	m

Sapindaceae	m	m	3	4	m	m	m	m	m				m	m	m	m		m	m	m	m

*Leucaena spp.*	m	m	m	m	m	m	m	m	m	m	m	m	m	m				m	m	m	m

*Julbernardia spp.*	m	m	m	m	m	m	m	m	m	m	m	m	m	m							

Acanthaceae								m	m	13	18	11	m	18	9	m	m	m	m	m	m

*Scheffleraspp.* (Ivy tree)	4	m	m	m	m	m	7	3	5	m	m	m									

*Chenopodium spp.* (black weed)	m	m	m	m	m	m	m	m	m	m	m	m									

*Onobrychis spp.*	m	m	m	m	m	m	m	m	m												

*Triumfetta spp.*					m	m	m	m	m	m	m	m								m	7

Apiaceae															m	m	m	4	4	m	m

Brassicaceae (crucifers)			m	m	m	m	m	m													

*Olea spp.*															m		23	m		m	m

*Coffea spp.*					m	m												18		13	m

*Vernonia spp.*					m	m	m	m													

*Taraxacum spp.*																		m	m	m	m

*Zea mays*																		m	m	m	m

Rhizophoraceae													29	42						23	

Ebenaceae							35										16				

*Prosopis spp.*								21	24												

Myrtaceae							m														m

*Eucalyptus spp.*							m														

*Trifoliumspp. *(clover types)																					m

Predominant (>45%), secondary pollen (16-45%), important minor pollen (3-15%) and minor pollen (m) (<3%).

## Data Availability

The data used in development of this article are available from the corresponding author upon request.
